# Zyxin promotes hepatocellular carcinoma progression via the activation of AKT/mTOR signaling pathway

**DOI:** 10.32604/or.2023.029549

**Published:** 2023-07-21

**Authors:** TIANYING CAI, JUNJIE BAI, PENG TAN, ZHIWEI HUANG, CHEN LIU, ZIMING WU, YONGLANG CHENG, TONGXI LI, YIFAN CHEN, JIAN RUAN, LIN GAO, YICHAO DU, WENGUANG FU

**Affiliations:** 1 Department of Hepatobiliary Surgery, Affiliated Hospital of Southwest Medical University, Luzhou, 646000, China; 2 Biobank, The Affiliated Hospital of Southwest Medical University, Luzhou, 646000, China; 3 Academician (Expert) Workstation of Sichuan Province, The Affiliated Hospital of Southwest Medical University, Luzhou, 646000, China; 4 Department of Medical Oncology, First Affiliated Hospital, School of Medicine, Zhejiang University, Hangzhou, 310000, China; 5 Department of Health Management, The Affiliated Hospital of Southwest Medical University, Luzhou, 646000, China

**Keywords:** Zyxin, Hepatocellular carcinoma, AKT/mTOR, Proliferation, Migration, Invasion

## Abstract

Hepatocellular carcinoma (HCC) is a common malignancy that is driven by multiple genes and pathways. The aim of this study was to investigate the role and specific mechanism of the actin-interacting protein zyxin (ZYX) in HCC. We found that the expression of ZYX was significantly higher in HCC tissues compared to that in normal liver tissues. In addition, overexpression of ZYX in hepatoma cell lines (PLC/PRF/5, HCCLM3) enhanced their proliferation, migration and invasion, whereas ZYX knockdown had the opposite effects (SK HEP-1, Huh-7). Furthermore, the change in the expression levels of ZYX also altered that of proteins related to cell cycle, migration and invasion. Similar results were obtained with xenograft models. The AKT/mTOR signaling pathway is one of the key mediators of cancer development. While ZYX overexpression upregulated the levels of phosphorylated AKT/mTOR proteins, its knockdown had the opposite effect. In addition, the AKT inhibitor MK2206 neutralized the pro-oncogenic effects of ZYX on the HCC cells, whereas the AKT activator SC79 restored the proliferation, migration and invasion of HCC cells with ZYX knockdown. Taken together, ZYX promotes the malignant progression of HCC by activating AKT/mTOR signaling pathway, and is a potential therapeutic target in HCC.

## Introduction

Liver cancer is one of the most common malignancies worldwide, and ranks sixth in terms of incidence and third in terms of mortality rate [1]. Hepatocellular carcinoma (HCC) is the most common type of liver cancer. Despite advances in radical surgery, targeted drugs, and interventional therapy, the mortality rate of HCC remains high due to frequent recurrence and metastasis. In addition, the clinical symptoms of HCC are insidious in initial stages and the disease progresses rapidly, which precludes early diagnosis and treatment, resulting in poor prognosis [2,3].

The occurrence and development of HCC is a complex biological process involving multiple mutations and dysregulation of various genes. Nevertheless, the exact molecular mechanisms underlying HCC progression remain unclear [4]. Recent studies have shown that the actin-interacting protein zyxin (ZYX), an LIM domain protein that translocates between the cytoplasm and nucleus, is a key oncogenic factor in glioblastoma and colorectal cancer [5,6]. ZYX is a component of focal adhesions (FA) between cells and the extracellular matrix, and is also involved in the organization and regeneration of the cytoskeleton [7]. There are no reports of its role in HCC so far. Therefore, our aim was to analyze the expression and biological functions of ZYX in HCC. We found that ZYX was significantly upregulated in the HCC tissues compared to the normal liver tissues, and enhanced the malignant potential of hepatoma cells by activating the oncogenic AKT/mTOR signaling pathway. Thus, ZYX is aberrantly expressed in HCC and promotes cancer progression via the AKT/mTOR pathway.

## Materials and Methods

### Bioinformatics analysis

The microarray dataset GSE14520 consisting of 22 HCC and 21 normal liver tissues specimens was downloaded from the Gene Expression Omnibus (GEO) database (http://www.ncbi.nlm.nih.gov/geo/) on the National Center for Biotechnology Information (NCBI) website. Expression matrix was formed with the raw counts of each RNA of each sample. The differentially expressed genes (DEGs) between HCC and normal liver tissues were screened using |log2FC| > 1 as the threshold. The expression level of the DEGs was log2(*+1)-transformed for further analysis, and the genes were visualized using heat maps and volcano plots. StarBase V3.0 and GEPIA were used to analyze the expression of zyx in HCC and normal liver tissues. The data of Human Protein Atlas was used to compare the overall survival of HCC patients demarcated on the basis of ZYX expression.

### Patients and specimens

Paired tumor and normal adjacent tissues were obtained from 17 HCC patients who underwent surgery at the Department of Hepatobiliary Surgery, Affiliated Hospital of Southwest Medical University. None of the patients had received adjuvant therapy. The fresh tissues were cut into 1-mm 3 pieces and immediately rinsed with cold saline. One portion of the tissues were snap-frozen in liquid nitrogen and stored at −80°C for molecular analyses, and the remaining were formalin-fixed and paraffin-embedded for *in situ* staining. Final diagnosis was confirmed by two experts based on the biopsy specimens. The patients provided written informed consent to participate in this study. The experiments involving human participants were reviewed and approved by the Clinical Trial Ethics Committee of Affiliated Hospital of Southwest Medical University (approval number: KY2019053).

### Cell culture

Human HCC cells, including BEL-7402, SK HEP-1, Huh7, MHCC97H, HCCLM3 and PLC/PRF/5, and the normal human liver cell line QSG-7701, were purchased from the cell bank of the Type Culture Collection of the Chinese Academy of Sciences (Shanghai, China). The cell lines were cultured in high-glucose DMEM (Catalog No. 12430054, Gibco, Shanghai, China) supplemented with 10% fetal bovine serum (FBS, Catalog No. ZQ500-A, Zhong Qiao Xin Zhou Biotechnology, Shanghai, China) and 1% penicillin and streptomycin (Catalog No. C0222, Beyotime, Shanghai Bio, Shanghai, China). All cells were grown in a humidified environment at 37°C under 5% CO_2_ and 95% air.

### Lentiviral transduction

Short hairpin RNA (shRNA) targeting human ZYX (shZYX) was purchased from Sangon Biotech (Shanghai, China) and the sequences were as follows: shZYX-1: 5′-CTGGGTCACAACCAAATCAAA-3′ and shZYX-2: 5′-CTTCCACATGAAGTGTTACAA-3′. A scrambled sequence (shNC) was included as the negative control. To generate lentiviruses, 293T cells were co-transfected with the shRNA plasmids and packaging plasmids (pLP1, pLP2 and pLP/VSVG). Cell supernatants were harvested 48 h later and used to infect hepatoma cells. After 48 h of infection, the medium was discarded and fresh medium supplemented with puromycin (4 μg/ml) was added. The cells were cultured for 48 h to screen for stable cell lines.

To construct the expression plasmid, the full sequence of ZYX was amplified by PCR using the following primers: forward 5′-acctccatagaagattctagaATG GCG GCC CCC CGC CCG-3′ and reverse 5′-ttcgaattcgctagctctagaTCA GGT CTG GGC TCT AGC AGT G-3′. The amplified ZYX sequence was then cloned into the pCDH vector (PCDH-ZYX), which was co-transfected into 293T cells along with the lentivirus packaging plasmids. HCC cells were infected with the lentivirus and stable clones were screened as described above.

### Cell viability assay

Cells were seeded in 96-well plates at the density of 2000 cells per well in 100 µL culture medium. After culturing the cells for 24, 48, 72 and 96 h, respectively, 10 µL Cell Counting Kit-8 reagent (CCK-8; Catalog No. C0038, Beyotime, Shanghai Bio, Shanghai, China) was added to each well. The cells were incubated for 2 h at 37°C, and the absorbance was measured at 450 nm using a microplate reader. In another experiment, the cells were respectively treated with 2.5 µg/mL MK2206 (Catalog No. SF2712-10 mM, Beyotime, Shanghai Bio, Shanghai, China) or 5 µg/mL SC79 (Catalog No. SF2730-10 mM, Beyotime, Shanghai Bio, Shanghai, China), and the viability was calculated as above.

### EdU staining

Cell proliferation was evaluated using the Click™ EdU-555 Cell Proliferation Kit (Catalog No. C0075S, Beyotime, Shanghai Bio, Shanghai, China). Briefly, the cells were seeded in 6-well plates and cultured till 70% confluence. After discarding the medium, 2 mL complete medium containing 10 μM EdU was added, and the cells were incubated for 2 h. The cells were washed thrice with PBS, fixed with 4% formaldehyde for 15 min at room temperature, washed again as described, and permeabilized with 0.3% Triton X-100 for 15 min. After three more washes with PBS, the cells were incubated with Click Additive Solution for 30 min, and the nuclei were counterstained with Hoechst-33342.

### Cell cycle and apoptosis assay

The suitably treated PLC/PRF/5 and SK HEP-1 cells were harvested, washed with PBS, and the cell count was adjusted to 1 × 105. After fixing overnight in 70% ethanol at 4°C, the samples were incubated with 10 mg/mL RNase and 1 mg/mL propidium iodide (Catalog No. C1052, Beyotime, Shanghai Bio, Shanghai, China) at 37°C for 30 min in the dark. The cell cycle distribution was determined on the basis of DNA content through flow cytometry (BD Biosciences). Apoptosis was evaluated using the Annexin V-FITC/PI Apoptosis Detection Kit (Catalog No. 40302ES20, YEASEN, Shanghai, China). Briefly, the PLC/PRF/5 and SK HEP-1 cells were digested with EDTA-free trypsin and collected in 1.5 mL EP tubes, washed with cold PBS, and centrifuged at 300 g for 5 min at 4°C. The cells were resuspended in 100 μL Binding Buffer for 5 min, and then stained with 5 μL Annexin V-FITC and 10 μL PI Staining Solution at room temperature for 10 min in the dark. The stained cells were resuspended in 400 μL Binding Buffer and analyzed by flow cytometry (BD Biosciences).

### Colony formation assay

The cells were seeded in 6-well plates at the density of 1000 cells per well, and cultured at 37°C for 10 days. The medium was discarded, and the colonies were washed with PBS, fixed with 4% paraformaldehyde and stained with crystal violet. The colonies were then photographed and counted.

### Scratch test

The cells were seeded in 6-well plates and cultured till 90% confluence. The monolayer was evenly scratched with a 200 µL pipette tip, and rinsed with PBS to remove the dislodged cells. Serum-free medium was added, and the scratched area was photographed under a microscope at 0, 24 and 48 h to monitor cell migration.

### Transwell assay

The migration of the suitably treated cells was tracked using transwell inserts in 24-well plates (Corning, Shanghai, China). The cells were resuspended in serum-free medium and seeded in the upper chambers of the inserts at the density of 5 × 104 cells/well, and the lower chamber were filled with complete medium containing 10% FBS. After 24 h incubation, the unmigrated cells remaining in the upper chamber were wiped off with a cotton swab, and migrated cells on the underside of the filter were fixed with 4% paraformaldehyde and stained with crystal violet. The number of migrated cells were then counted under a microscope. For the invasion assay, membranes were coated with Matrigel (BD Biosciences, Shanghai, China) and the other steps were the same as in the migration assay.

### Tumor xenografts

Ten 4-weeks-old male BALB/c nude mice were purchased from Chongqing Tengxin Biotechnology Company (Chongqing, China) and housed in a temperature and humidity-controlled laboratory animal room. The mice were randomly divided into the control and ZYX-knockdown groups, and respectively injected subcutaneously with 5 × 105 SK HEP-1-shNC or SK HEP-1-shZYX-2 cells in 0.1 mL serum-free into the right axilla. The mice were euthanized after 24 days, and the tumors were removed and weighed. The length and width of the tumors were measured, and the volume was calculated as length × (width) 2/2. Part of the tumor tissues from each group were fixed in 4% paraformaldehyde for staining, and the remaining were used for protein extraction. Animal experiments were reviewed and approved by the Southwest Medical University Laboratory Animal Ethics Committee (approval number: 20211119-062).

### Quantitative real-time PCR (qRT-PCR)

Total RNA was extracted from hepatoma cells using RNA-easy Isolation Reagent (Catalog No. R701-01, Vazyme, Nanjing, Jiangsu, China) according to the manufacturer’s instructions, and reverse transcribed using Master Mix (Cat. Q712-02, Vazyme, Nanjing, Jiangsu, China). The reaction mix for qRT-PCR included 2 µL sample cDNA, 0.4 µL each forward and reverse primers, 7.2 µL enzyme-free water and 10 µL Taq Pro Universal SYBR qPCR Master Mix in a total volume of 20 µl. The expression of each gene was normalized to β-actin expression. The primer sequences were as follows: zyx forward 5′-TCTCCCGCGATCTCCGTTT-3′ and reverse 5′-CCGGAAGGGATTCACTTTGGG-3′; β-actin forward 5′-ATCGTGCGTGACATTAAGGAGAAG-3′ and reverse 5′-AGGAAGGAAGGCTGGAAGAGTG-3′.

### Western blotting

The tissues and cells were homogenized using RIPA lysis buffer (Catalog No. P0013B, Beyotime, Shanghai, China) supplemented with protease inhibitors (Catalog No. P1005, Beyotime, Shanghai, China) and phosphoprotease inhibitors (Catalog No. P1081, Beyotime, Shanghai, China). The samples were placed on ice for 1 h with intermittent mixing. The lysates were centrifuged at 13000 rpm for 10 min at 4°C, and the protein content in the supernatants was measured using a BCA protein assay kit (Catalog No. P0012, Beyotime, Shanghai, China). Equal amounts of protein per sample were separated by 10% SDS-PAGE and transferred to polyvinylidene fluoride (PVDF) membranes. After blocking with 5% non-fat milk in Tris-buffered saline containing Tween 20 (TBST) for 1 h, the membranes were washed thrice with TBST and incubated overnight with primary antibodies at 4°C. The following day, the membranes were washed thrice with TBST for 10 min each time, incubated with the secondary antibody for 1 h at room temperature, and washed thrice with TBST again. The positive bands were developed using an enhanced chemiluminescence detection system (Catalog No. P0018FS, Beyotime, Shanghai, China).

### Hematoxylin–eosin (HE) and immunohistochemistry (IHC) staining

The paraffin-embedded tissues were cut into 5 µm-thick slices, dewaxed in xylene, and rehydrated with graded alcohol (100%, 100%, 95%, 80% and 70%). HE staining was performed using a commercial kit (Catalog No. C0105, Beyotime, Shanghai, China).

For IHC, the cleared and rehydrated sections were heated in 0.01M citrate buffer (pH 6) for 20 min in the microwave for antigen retrieval, and then treated with 3% hydrogen peroxide for 10 min to quench peroxidase activity. The sections were incubated overnight with primary antibodies specific for ZYX (1:100), MMP-2 (1:200), and Ki-67 (1:2000) at 4°C. The secondary antibody was added the following day, and the slides were incubated at room temperature for 1 h. After washing with PBS, the sections were stained with diaminobenzidine and then counterstained with hematoxylin. The stained sections were dehydrated and sealed, and observed under a microscope (Olympus, Chengdu, Sichuan, China).

### Statistical analysis

Data were expressed as mean ± standard deviation of three independent experiments. Inter-group differences were assessed for significance using the two-tailed Student’s *t* test, and *p* < 0.05 was considered statistically significant.

## Results

### ZYX is overexpressed in HCC tissue

**Figure 1:**
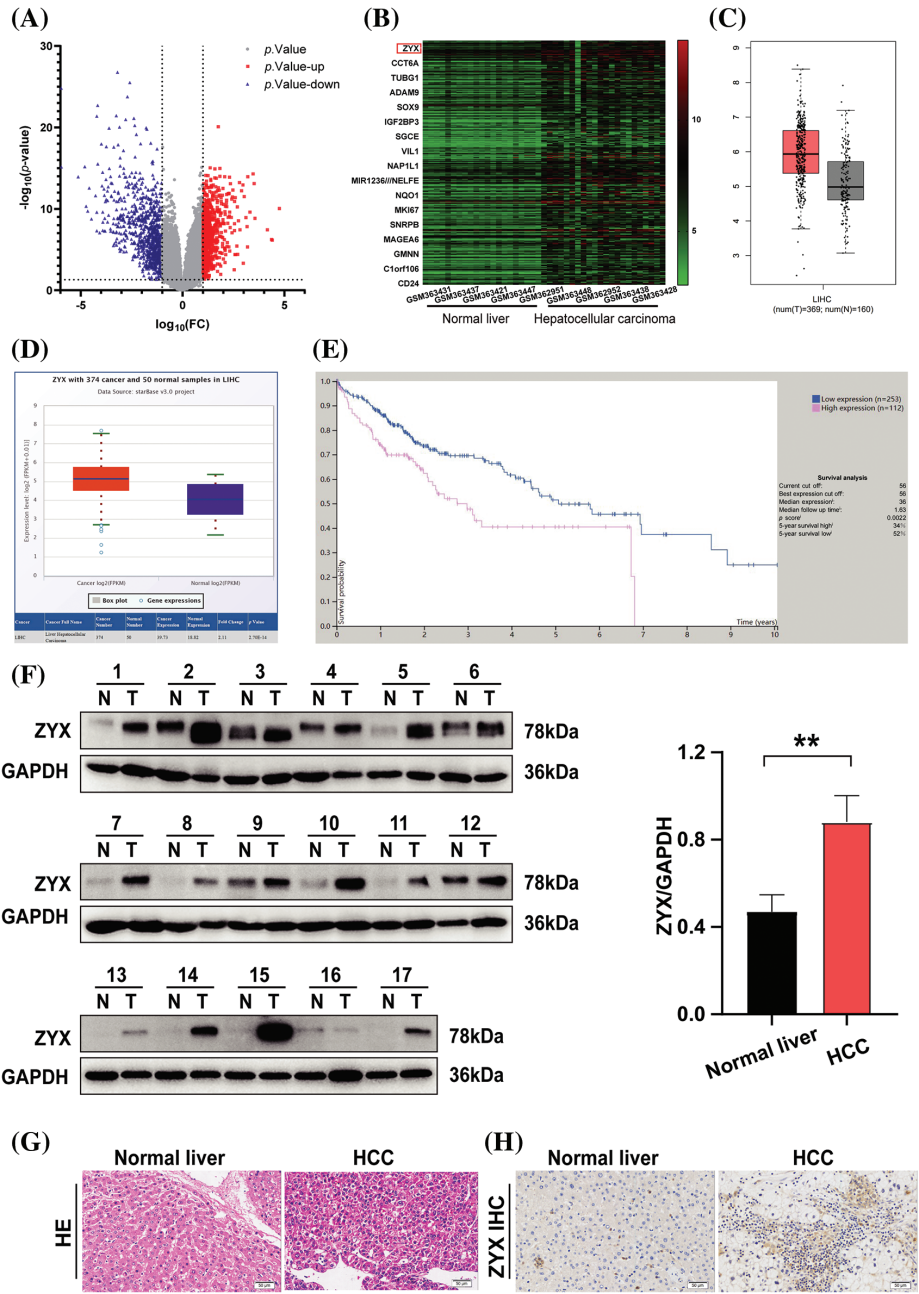
ZYX is overexpressed in HCC tissue. (A) Volcano plot visualizing the all differentially expressed genes in GSE14520. (B) Heatmap was used to show the unsupervised clustering of the top 300 up-regulated genes. (C) The result of analysis from GEPIA indicated that the overexpression of ZYX in HCC samples (*p < 0.01). (D) The result of analysis on StarBase V3.0 revealed that the higher expression of ZYX in HCC patients compared with that in normal (*p* = 2.70e-14). (E) The Human Protein Atlas suggests that HCC patients with low ZYX have better the overall survival rate than the HCC patients with high ZYX. (F) ZYX is more expressed in cancer tissues of HCC patients than in normal tissues. **p < 0.01 (N = 17). (G) H&E staining in cancer tissue and control tissues from HCC patients. (H) Immunohistochemistry analysis of ZYX in cancer tissue and control tissues from HCC patients.

Analysis of the GSE14520 dataset revealed that 710 genes were significantly up-regulated and 896 genes were down-regulated in HCC tissues compared to normal liver tissues (Fig. 1A). Furthermore, ZYX was one of the most significantly up-regulated genes in HCC (Fig. 1B), which was also confirmed by StarBase V3.0 and GEPIA (Figs. 1C and 1D). According to the overall survival data from the Human Protein Atlas, HCC patients with low ZYX expression had better prognosis compared to those with high ZYX expression (Fig. 1E). To confirm these findings, we analyzed paired tumor and adjacent normal tissue samples from 17 patients with pathologically diagnosed HCC. As shown in Fig. 1F, ZYX protein level was markedly higher in the HCC tissues compared to the normal liver tissues. *In situ* staining of the tissues clearly differentiated the pathological features of HCC from normal liver parenchyma (Fig. 1G), and also confirmed the higher expression of ZYX in the former (Fig. 1H).

### Construction of human hepatoma cell lines with stable overexpression or knockdown of ZYX

**Figure 2:**
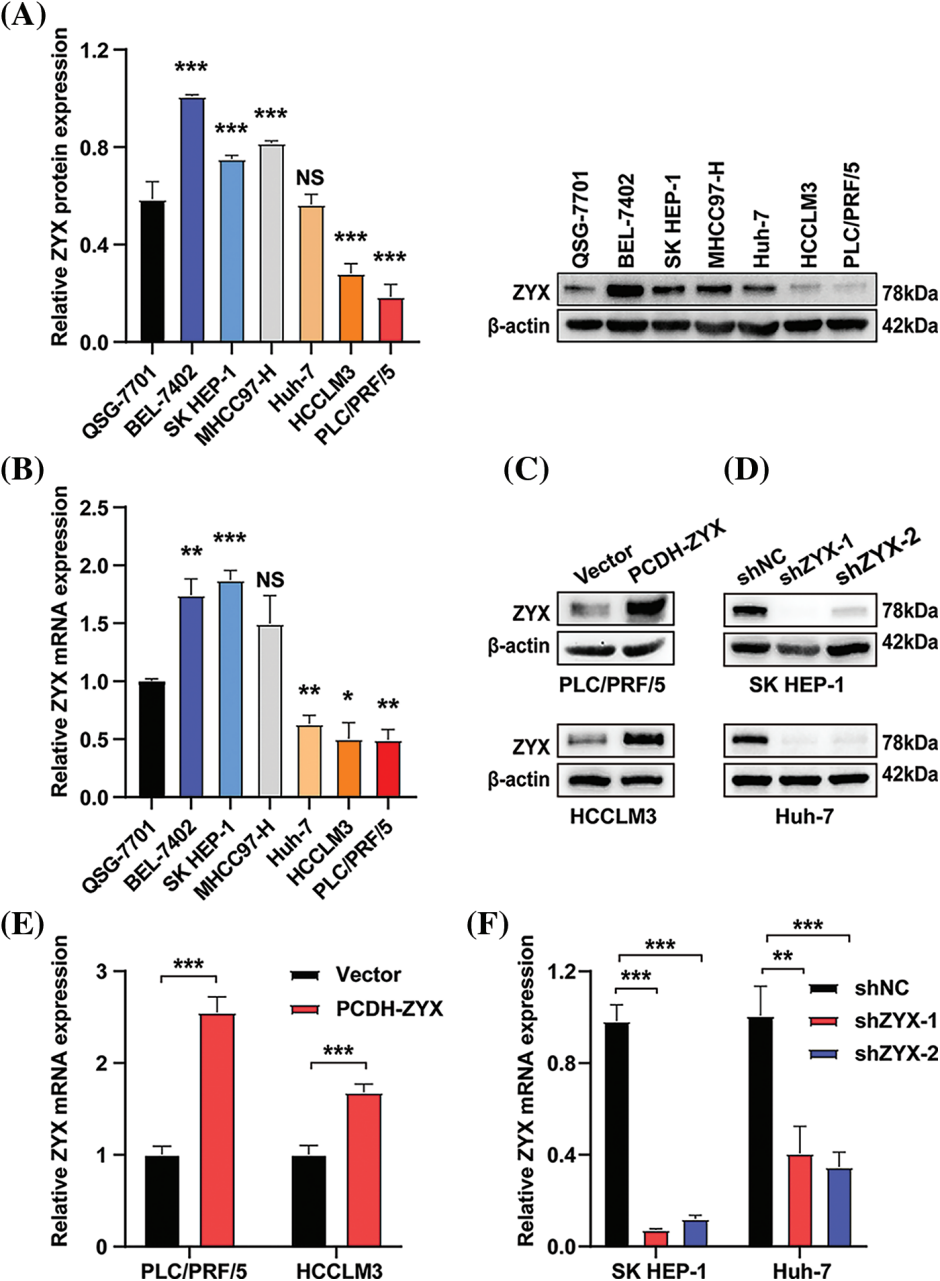
Construction of human hepatoma cell lines with stable overexpression or knockdown of ZYX. (A) ZYX protein levels in different human HCC cell lines (N = 3). (B) mRNA levels of zyx in different human HCC cell lines (N = 3). (C) PLC/PRF/5 and HCCLM3 cells was transfected with PCDH-ZYX plasmid, and Vector was used as a control. ZYX was detected using western blot (N = 3). (D) The zyx was detected using qRT-PCR (N = 3). (E) SK HEP-1 and Huh7 cells were transfected with shZYX-1 or shZYX-2 sequences, and shNC was used as a control. ZYX was detected using western blot (N = 3). (F) The zyx was detected using qRT-PCR. Data are the mean of three experiments (mean ± SD) (N = 3). NS *p* > 0.05, *p < 0.05, **p < 0.01, ***p < 0.001.

Compared to normal human hepatocytes, ZYX protein was upregulated in hepatoma cell lines including BEL-7402, SK HEP-1, and MHCC97H, but was expressed at lower levels in the HCCLM3 and PLC/PRF/5 cells (Fig. 2A). Similar results were obtained with qRT-PCR (Fig. 2B). Therefore, we constructed stable PLC/PRF/5 and HCCLM3 lines overexpressing ZYX (Figs. 2C and 2D), and SK HEP-1 and Huh7 cell lines with ZYX knockdown (Figs. 2E and 2F). The overexpression and knockdown of ZYX in the respective cell lines were confirmed by qRT-PCR and western blotting.

### ZYX promoted HCC cell proliferation and cell cycle transition in vitro

**Figure 3:**
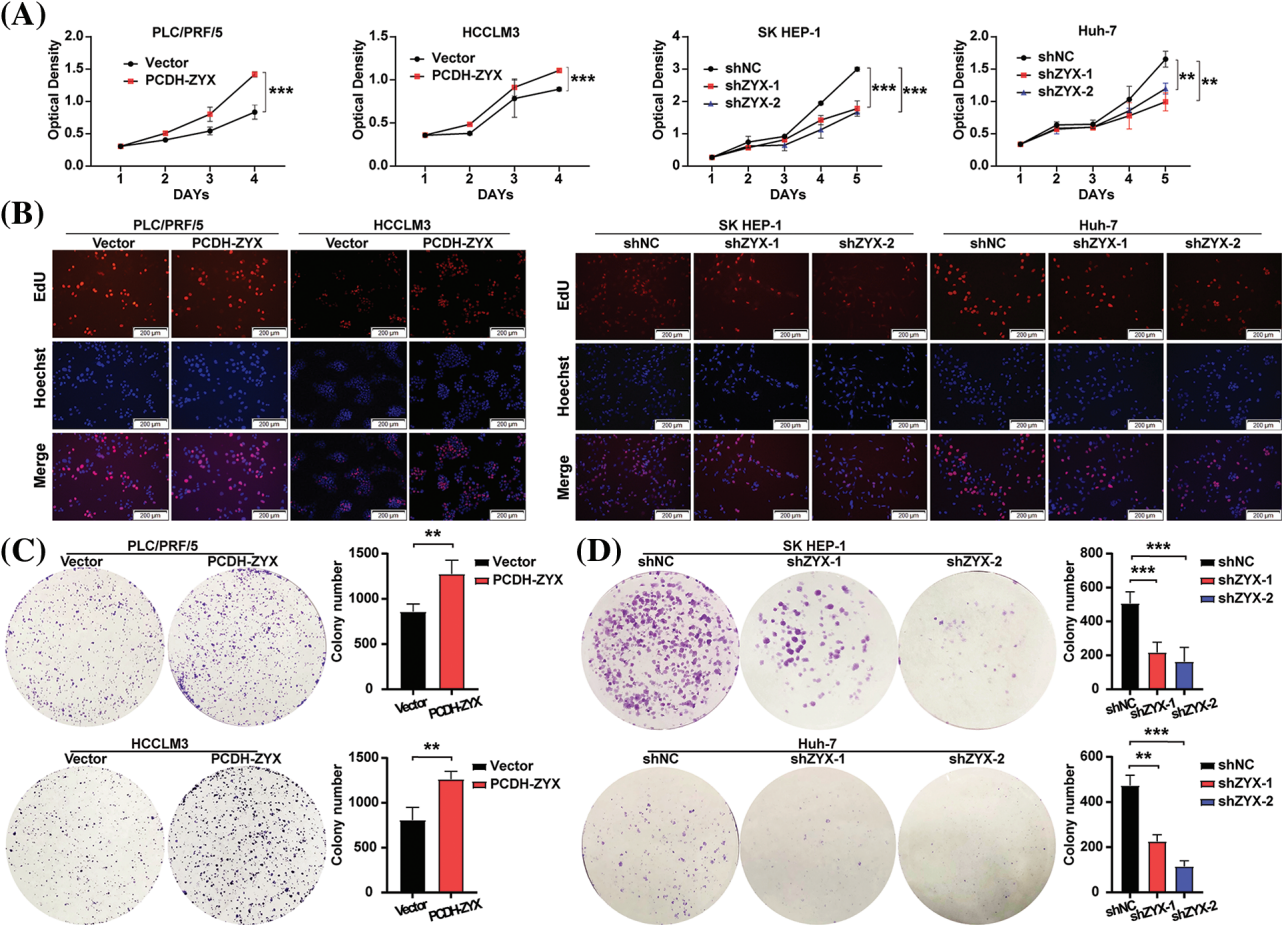
ZYX affects HCC cell proliferation and colony forming *in vitro*. (A) Effects of ZYX overexpression or knockdown on proliferation rate by HCC cells (N = 3). (B) Edu staining showed the effect of ZYX overexpression or knockdown on proliferation by HCC cells. (C) Effects of ZYX overexpression in PLC/PRF/5 and HCCLM3 on colony formation by HCC cells (N = 3). (D) Effects of ZYX knockdown in SK HEP-1 on colony formation by HCC cells (N = 3). Data are the mean of three experiments (mean ± SD). **p < 0.01, ***p < 0.001.

**Figure 4:**
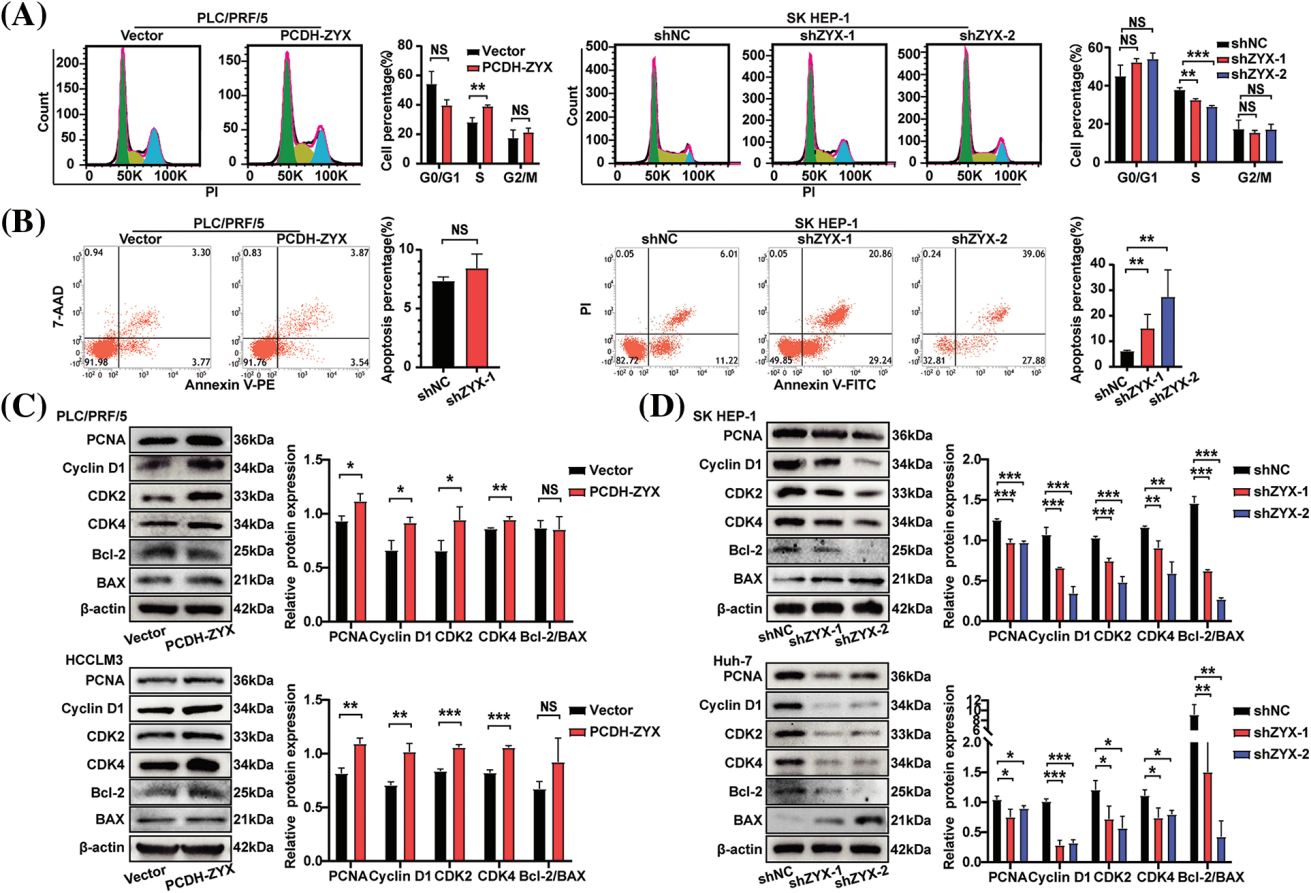
ZYX affects the expression of proliferation and cell cycle related proteins in hepatoma cells and HCC cell colony formation *in vitro*. (A) Cell cycle analysis of using flow cytometry after ZYX overexpression or knockdown in HCC cells (N = 3). (B) Cell apoptosis analysis of using flow cytometry after ZYX overexpression or knockdown in HCC cells (N = 3). (C) ZYX overexpression up-regulated PCNA, Cyclin D1, CDK2, CDK4, Bcl-2 and BAX (N = 3). (D) ZYX knockdown down-regulated PCNA, Cyclin D1, CDK2, CDK4, Bcl-2 and BAX (N = 3). Data are the mean of three experiments (mean ± SD). NS *p* > 0.05, *p < 0.05, **p < 0.01, ***p < 0.001.

ZYX overexpression significantly increased the viability of HCC cells compared to that of the control group, whereas knocking down ZYX had the opposite effect (Fig. 3A). In addition, EdU incorporation assay showed that HCC cells overexpressing ZYX had higher proliferation rates compared to the control, whereas ZYX knockdown slowed proliferation (Fig. 3B). Likewise, high ZYX expression significantly increased the colony forming capacity of HCC cells, while inhibition of ZYX decreased the number and size of colonies (Figs. 3C and 3D). Consistent with this, the proportion of HCC cells entering the S phase increased with ZYX overexpression, whereas silencing ZYX significantly decreased the proportion of proliferative cells in the S phase (Fig. 4A). This indicated that ZYX promotes the proliferation of HCC cells by accelerating progression through the cell cycle. Interestingly, overexpression of ZYX did not seem to have a significant effect on the apoptosis rates of HCC cells, although the proportion of both apoptotic and necrotic cells increased significantly after ZYX knockdown (Fig. 4B). At the molecular level ZYX increased, the expression of proteins related to cell proliferation and cell cycle such as Proliferating Cell Nuclear Antigen (PCNA), cyclin D1, cyclin-dependent kinase 2 (CDK2) and CDK4, but did not alter the expression of the anti-apoptosis protein Bcl-2 and pro-apoptosis protein BAX significantly (Fig. 4C). On the other hand, knockdown of ZYX inhibited the cyclin proteins downregulated Bcl-2 and upregulated BAX (Fig. 4D).

### ZYX accelerated migration and invasion of HCC cells in vitro

**Figure 5:**
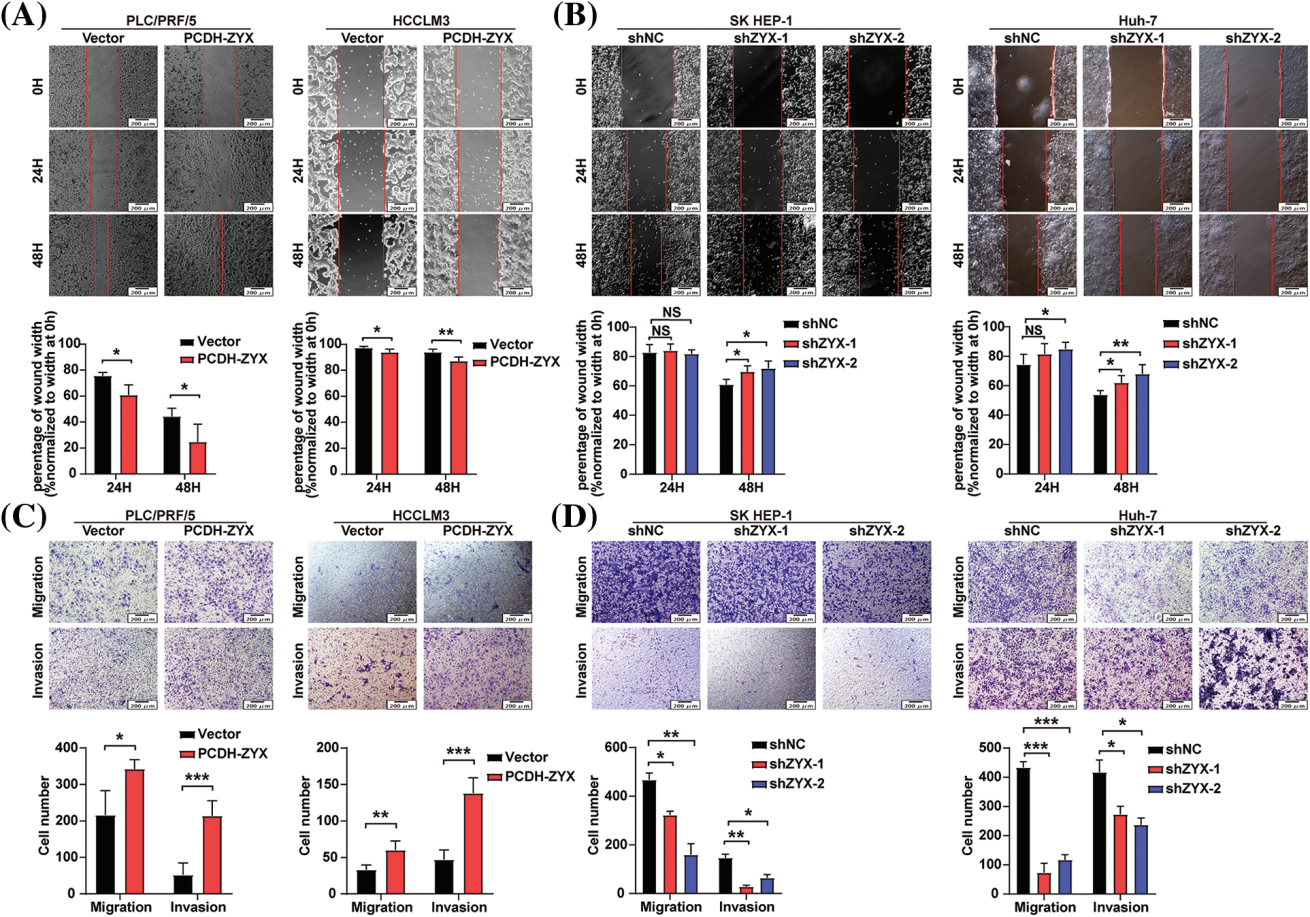
ZYX accelerated migration and invasion of HCC cells *in vitro*. (A) Scratch test to detect the cell movement of PLC/PRF/5 and HCCLM3 when ZYX was overexpressed (N = 3). (B) Scratch test to detect the cell movement of SK HEP-1 and Huh-7 cells when ZYX was inhibited (N = 3). (C) Transwell experiment to detect the migration and invasion of PLC/PRF/5 cells and HCCLM3 cells under the high ZYX (N = 3). (D) Transwell experiment to detects the migration and invasion of SK HEP-1 cells and Huh-7 cells under the low ZYX. Data are the mean of three experiments (mean ± SD) (N = 3). Data are the mean of three experiments (mean ± SD). NS *p* > 0.05, *p < 0.05, **p < 0.01, ***p < 0.001.

**Figure 6:**
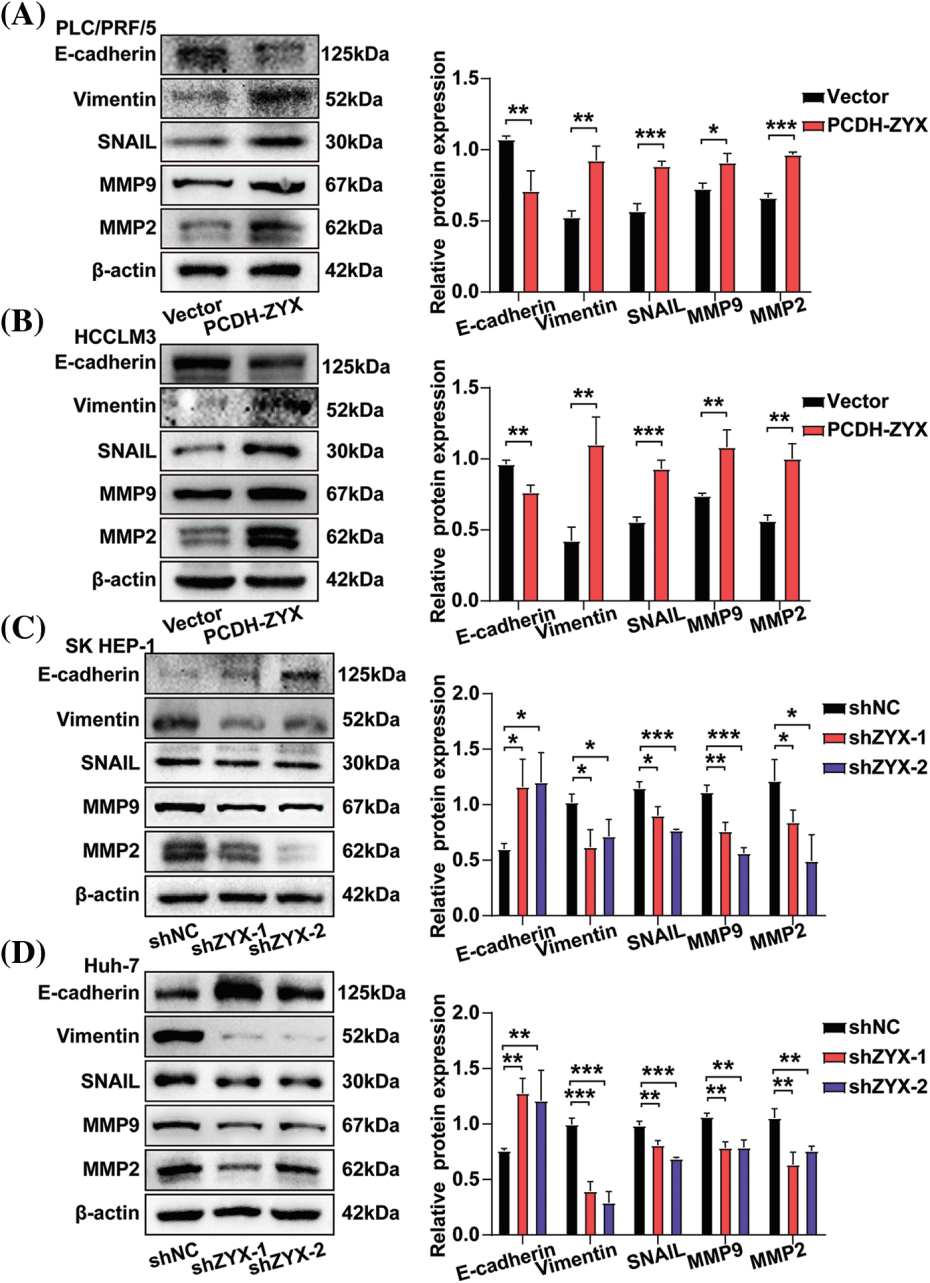
ZYX affects the expression of migration and invasion related proteins in hepatoma cells. (A, B) ZYX overexpression up-regulated the expressions of E-cadherin, Vimentin, Snail, MMP-9 and MMP-2 (N = 3). (C, D) ZYX expression inhibition down-regulated the expressions of E-cadherin, Vimentin, Snail, MMP-9 and MMP-2 (N = 3). Data are the mean of three experiments (mean ± SD). *p < 0.05, **p < 0.01, ***p < 0.001.

The effect of ZYX on the migratory and invasive abilities of HCC cells were evaluated by the wound healing and transwell assays. In the scratch test, HCC cells overexpressing ZYX resulted in faster healing of the wound area (Fig. 5A), indicating enhanced cell migration. On the other hand, ZYX knockdown significantly slowed the wound healing rate (Fig. 5B). In the transwell experiments, ZYX-overexpressing HCC cells showed stronger migratory and invasive abilities (Fig. 5C), while knocking down ZYX significantly attenuated both migration and invasion rates compared to that of control cells (Fig. 5D). Furthermore, high levels of ZYX significantly increased the expression of mesenchymal proteins such as Vimentin, MMP-9, MMP-2 and Snail, and decreased that of the epithelial marker E-cadherin. ZYX silencing led to opposite trends in the expression levels of the above proteins (Fig. 6). Based on these findings, we concluded that ZYX promotes the migration and invasion of HCC cells by promoting the epithelial-mesenchymal transition (EMT).

### ZYX regulated the AKT/mTOR signaling pathway in hepatoma cells

**Figure 7:**
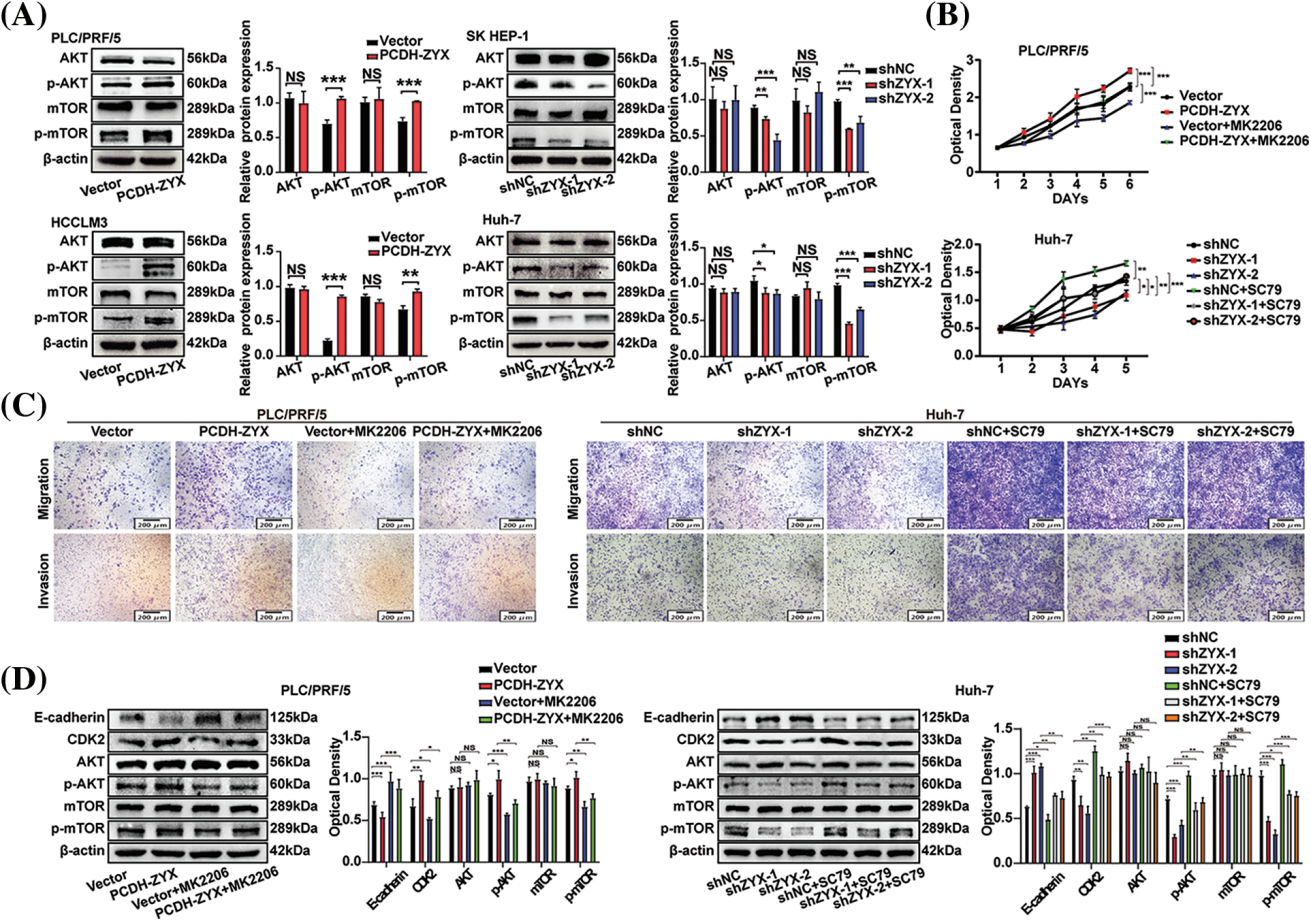
ZYX regulated the AKT/mTOR signaling pathway in hepatoma cells. (A) Overexpression of ZYX up-regulated the phosphorylation levels of AKT and mTOR, but the contents of AKT and mTOR did not change significantly. Decreased ZYX inhibits the phosphorylation of AKT and mTOR, and the content of AKT and mTOR has no significant change (N = 3). (B) AKT inhibitor MK2206 counteracted the promotion of PLC/PRF/5 proliferation caused by high ZYX, and AKT activator SC79 promoted Huh-7 cell proliferation when ZYX decreased (N = 3). (C) AKT inhibitor MK2206 counteracted the promotion of PLC/PRF/5 migration and invasion caused by high ZYX, and the AKT activator SC79 counteracted the inhibitory effect of Huh-7 migration and invasion caused by low ZYX. (D) Effects of AKT inhibitor MK2206 and AKT activator SC79 on invasion, cyclin and the expressions of AKT/mTOR pathway-related protein, respectively (N = 3). Data are the mean of three experiments (mean ± SD). NS *p* > 0.05, *p < 0.05, **p < 0.01, ***p < 0.001.

The AKT/mTOR signaling axis is an important regulatory pathway in the initiation and development of HCC. As expected, ZYX overexpression significantly upregulated the levels of phosphorylated AKT and mTOR in HCC cells, whereas ZYX silencing had the opposite effect. However, the levels of total AKT and mTOR did not change significantly (Fig. 7A). To determine whether the oncogenic effects of ZYX are mediated by the AKT/mTOR pathway, we treated the ZYX-overexpressing and ZYX-knockdown cells with the AKT inhibitor MK2206 and the AKT activator SC79 respectively. While MK2206 significantly decreased the proliferation rates of HCC cells overexpressing ZYX, SC79 enhanced the proliferative capacity of HCC cells with ZYX knockdown (Fig. 7B). Furthermore, MK2206 also reduced the migration and invasion rates of ZYX-overexpressing HCC cells, whereas SC79 restored these properties in the ZYX-silenced HCC cells (Fig. 7C). Finally, MK2206 and SC79 respectively decreased and increased p-AKT/mTOR levels, and similarly altered the expression of E-cadherin and CDK2 proteins (Fig. 7D).

### ZYX promoted the growth of HCC xenograft in vivo

**Figure 8:**
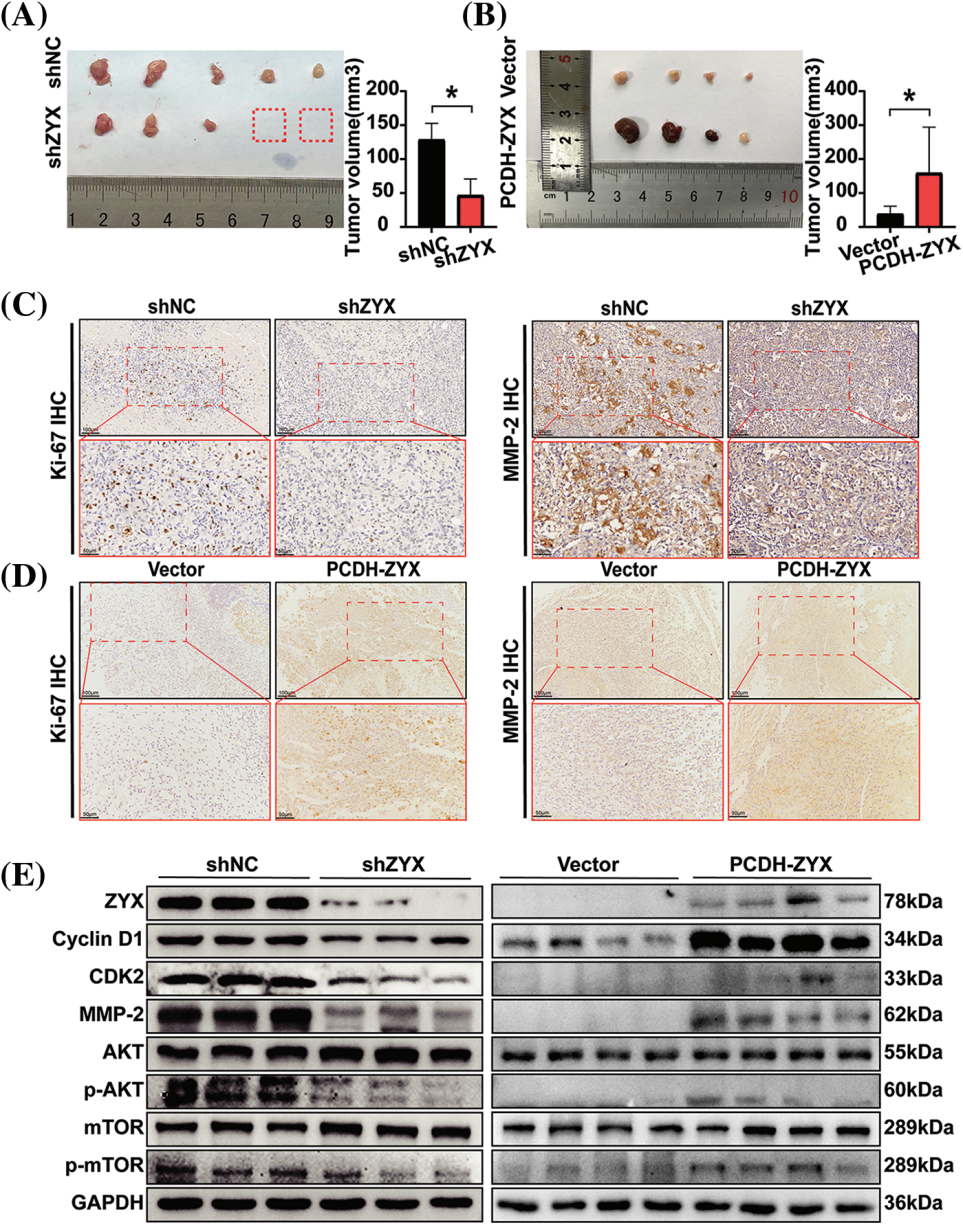
ZYX affects the proliferation of HCC cells *in vivo*. (A) Decreased ZYX leads to slower tumor growth and lower tumorigenesis rate *in vivo* (N = 5). (B) ZYX overexpression promotes tumor growth *in vivo* (N = 4). Data are the mean of multiple experiments (mean ± SD). *p < 0.05. (C) IHC analysis of Ki-67 and MMP-2 in tissue samples of xenograft model with ZYX interference. (D) IHC analysis of Ki-67 and MMP-2 in tissue samples of ZYX overexpressed xenograft model. (E) The expression of cycle-associated proteins and AKT/mTOR pathway-related protein was changed with the abnormal expression of ZYX *in vivo*.

The *in vitro* findings were further validated by establishing HCC xenografts in mouse models. The HCC cells with ZYX knockdown formed significantly smaller tumors with lower growth rate compared to the tumors derived from control cells (Fig. 8A). In contrast, overexpression of ZYX accelerated tumor growth *in vivo* (Fig. 8B). Furthermore, immunostaining of the tumor tissues showed lower expression of Ki-67 and MMP-2 in the shZYX group compared to that in the control group, as opposed to increased expression in the PCDH-ZYX group (Fig. 8C). Likewise, compared to the control group, cyclin D1, CDK2, MMP-2, p-AKT and p-mTOR protein levels were significantly decreased in the shZYX tumors, and showed an increase in ZYX-overexpressing tumors (Fig. 8D). Taken together, ZYX promoted the growth of HCC xenograft *in vivo* by enhancing proliferation of the tumor cells.

## Discussion

HCC is a common malignancy characterized by high morbidity and mortality rates, and poor prognosis. It is often the sequel to hepatitis and liver cirrhosis, and is driven by a complex network of genes [8,9]. Therefore, it is imperative to explore the molecular mechanisms underlying the progression of HCC in order to identify novel diagnostic biomarkers and therapeutic targets. We found that ZYX functions as an oncogene in HCC, and is up-regulated in HCC tissues and correlates with poor prognosis. Furthermore, forced expression of ZYX in HCC cells enhanced their malignant potential, whereas its knockdown had the opposite effects.

Cyclin [10–12] and CDKs [13–15] drive the cell cycle, and are frequently dysregulated during cancer progression. The transition from the G1 checkpoint to the S phase is mediated by the CDK4-cyclin D and CDK2-cyclin E/A complexes. The overexpression of ZYX significantly increased the levels of PCNA, cyclin D1, CDK2 and CDK4 in the HCC cells, indicating that ZYX promotes the proliferation of HCC cells by accelerating G1/S progression [16–19]. This hypothesis was consistent with the higher rates of EdU incorporation and colony formation seen in the ZYX-overexpressing cells. In contrast, ZYX knockdown had an inhibitory effect on the proliferative capacity of HCC cells. Interestingly, high ZYX expression did not significantly affect the apoptosis rates of HCC cells. On the other hand, ZYX knockdown increased apoptosis and necrosis rates, and reduced the Bcl-2/BAX ratio. We surmised that HCC cells cannot sustain the rapid proliferation rates in the absence of ZYX, resulting in apoptosis and necrosis. Thus, ZYX is an effective therapeutic target in HCC, and its inhibition can trigger apoptotic cell death. However, the mechanisms underlying apoptosis and necrosis are highly complex, and the same gene may not only promote but also inhibit apoptosis [20]. Therefore, the exact role of ZYX in the apoptosis of HCC cells requires further experimental verification.

High ZYX expression also increased the migration and invasion of HCC cells by regulating the EMT-related proteins including E-cadherin, Vimentin and Snail [21–23], as well as the extracellular proteins MMP-2 and MMP-9 [24–26]. Previous studies have established the key role of EMT and the MMP family of proteins in cancer cell migration, invasion and metastasis [27,28]. Consistent with the *in vitro* results, ZYX overexpression enhanced the tumorigenicity of HCC cells *in vivo*, thereby increasing the growth rate and size of the xenografts. On the other hand, inhibition of ZYX reduced the tumorigenicity and growth rate of HCC cells. Based on these findings, we surmised that ZYX is an oncogene and a promising therapeutic target in HCC.

Several studies have shown that ZYX is upregulated in various cancers, including glioblastoma multiforme [29], colorectal cancer [30], oral squamous cell carcinoma [31] and melanoma [32]. ZYX promotes the development of stable FA from early focal complexes (FX) that consist of integrin and focal adhesion kinase (FAK) [33]. The formation of FA mediates cell adhesion to the extracellular matrix, and modulates integrin-mediated signaling pathways related to cell motility, migration, invasion, survival, immunosuppression and apoptosis [34]. Furthermore, ZYX may regulate key cancer-related signaling pathways [35], such as the Hippo [36,37] and the Jun signaling pathways [30]. The AKT/mTOR pathway regulates cellular glucose metabolism, growth and reproduction [38], and is a recognized oncogenic driver [39] which mediates metabolic imbalance and cell cycle progression to enhance the survival and growth of cancer cells [40,41]. In addition, activation of AKT/mTOR signaling pathway inhibits apoptosis of cardiomyocytes [42]. We found that ZYX overexpression activated the AKT/mTOR pathway, whereas its knockdown inhibited the same. Furthermore, the AKT inhibitor MK2206 and activator SC79 respectively counteracted the effects of ZYX overexpression and ZYX knockdown on the proliferation, migration, and invasiveness of HCC cells.

Taken together, our findings indicate that ZYX promotes HCC progression by activating the AKT/mTOR signaling pathway. In fact, this is the first study to show a relationship between ZYX and the AKT/mTOR signaling pathway in HCC. However, although we detected changes in the levels of phosphorylated AKT and mTOR, we did not analyze whether ZYX directly phosphorylates AKT/mTOR, or activates the pathway through other mechanisms. Our study provides new ideas and possibilities for the treatment of HCC. However, our research is still far from clinical application, and more and deeper mechanism research and clinical application research are needed. In conclusion, our findings provide new insights into the molecular mechanisms driving HCC progression, and establish ZYX as a novel prognostic biomarker and therapeutic target for HCC.

## Conclusion

In this study, we aimed to investigate the carcinogenic effect of ZYX on human HCC. We found that ZYX is highly expressed in human HCC tissues, which mainly promotes the phosphorylation and activation of AKT/mTOR signaling pathway, regulates the expression of downstream related proteins, and thus plays a role in promoting the proliferation, migration and invasion of human HCC cells, mediating the growth of HCC tumors and disease progression. It may be a new effective therapeutic target for HCC.

## Data Availability

The datasets used and/or analyzed during this study are available from the corresponding author upon reasonable request.
